# Pharmacokinetics, toxicity, and cytochrome P450 modulatory activity of plumbagin

**DOI:** 10.1186/s40360-016-0094-5

**Published:** 2016-11-14

**Authors:** Wiriyaporn Sumsakul, Tullayakorn Plengsuriyakarn, Kesara Na-Bangchang

**Affiliations:** 1 Graduate Program in Biomedical Sciences, Faculty of Allied Health Sciences, Thammasat University, Pathumthani, Thailand; 2 Graduate Program in Bioclinical Sciences, Chulabhorn International College of Medicine, Thammasat University, Pathumthani, Thailand; 3 Center of Excellence in Pharmacology and Molecular Biology of Malaria and Cholangiocarcinoma, Thammasat University, Pathumthani, Thailand

**Keywords:** Plumbagin, Antimalarial drug, Pharmacokinetics, Toxicity, Cytochrome P450, Metabolic drug interaction

## Abstract

**Background:**

The antimalarial activity of plumbagin (5-hydroxy-2-methyl-1,4-naphthoquinone), a naturally occurring naphthoquinone widely distributed in the Plumbaginaceae family has previously been demonstrated in vitro (good activity) and in vivo (weak activity). The aim of the study was to investigate the pharmacokinetic profile following a single oral dosing to explain inconsistency of results of the in vitro and in vivo antimalarial activities. In addition, toxicity profiles and potential of modulation of cytochrome P450 enzymes (CYP1A2 and CYP3A11) were also investigated.

**Methods:**

The pharmacokinetics and toxicity of plumbagin were investigated in rats. The propensity of plumbagin to modulate the mRNA expression and activities of the two inducible forms of hepatic drug metabolizing enzyme cytochrome P450 (CYP450), i.e., CYP1A2 and CYP3A11, was investigated using microsomes prepared from mouse livers.

**Results:**

Acute and subacute toxicity tests indicate low toxicity of plumbagin with maximum tolerated doses of 150 (single oral dose) and 25 (daily doses for 28 days) mg/kg body weight, respectively. The pharmacokinetic profile of plumbagin following a single oral dose of 100 mg/kg body weight suggests that delayed absorption and short residence time (median values of time to maximal concentration and elimination half-life = 9.63 and 5.0 h, respectively) in plasma. Plumbagin did not modulate mRNA expression and activities of CYP1A2 and CYP3A11.

**Conclusions:**

Plumbagin was well tolerated following oral dose administration in rats. Pharmacokinetic property of this compound may be a limiting factor that explains the weak antimalarial activity of plumbagin observed in animal models. Potential metabolic interaction with co-administered drugs that are metabolized by CYP1A2 or CYP3A11 are unlikely.

## Background

Chemotherapy remains the mainstay for malaria control in the absence of a suitable vaccine treatment. Natural products including medicinal plants have been well demonstrated as promising sources of effective antimalarial drugs [1]. Plumbagin (5-hydroxy-2-methyl-1,4-naphthoquinone, a naturally occurring naphthoquinone widely distributed in the Plumbaginaceae family, has been reported to possess a wide spectrum of biological and pharmacological properties including activities against malaria, leishmania and trypanosome parasites, as well as against virus, cancers, and bacteria [2]. Promising antimalarial activities of both plumbagin and the crude ethanolic extract of *Plumbago indica* Linn. (root) have been demonstrated in vitro and in vivo [3, 4]. In the in vitro model, the extract exhibited good to moderate antimalarial activity (class III antimalarial activity) with IC_50_ (concentration producing 50 % growth inhibition) of 3 and 6.2 μg/mL, against K1 chloroquine-resistant and 3D7 chloroquine-sensitive *P. falciparum* clones, respectively [3]. Based on its relatively high selectivity index (SI = 44.7 and 21.6, respectively), it appears to be safe for human used for treatment of malaria. The active constituent plumbagin also shows promising antimalarial activity with median IC_50_ values against K1 and 3D7 *P. falciparum* clones of 370 and 580 nM, respectively. The mean IC_50_ values of the standard antimalarial drugs artesunate, mefloquine, quinine and chloroquine against K1 and 3D7 *P. falciparum* clones are 1.5 vs. 2 nM, 12 vs. 10 nM, 133- vs. 52 nM and 130 vs. 8 nM, respectively [5]. The activity against *P. falciparum* enzyme succinate dehydrogenanse (SDH) including parasite growth has been shown to be inhibited to 50 % by plumbagin at inhibitory concentrations of 5 and 0.27 mM, respectively [6]. In the in vivo model, plumbagin at the dose of 25 mg/kg body weight given for 4 days however, exhibited only weak to antimalarial activity with regard to the inhibitory activity on the reduction of parasitemia and the prolongation of survival time [4].

The inconsistencies of the observed antimalarial activity of plumbagin in the in vitro and in vivo models have been partly clarified in the following series of investigation with particular attention on the pharmacokinetic factor. Permeability of the compound across the human colon epithelial cell monolayer Caco-2 has been shown to be moderate and the transport mechanism is likely to be a passive transport [7]. With the application of single-photon emission computed tomography/computed tomography (SPECT/CT) imaging system and radiochemical analysis, blood kinetics and tissue distribution of radiolabeled plumbagin (^99m^Technetium-plumbagin complex) was investigated in healthy and *P. berghei*-infected mice [8]. The labeled complex distributed to all organs of both healthy and infected mice but with high intensity in liver, followed by lung, stomach, large intestine, and kidney. Accumulation in spleen was markedly noticeable in the infected mice. Plumbagin-labeled complex was rapidly cleared from blood with short elimination half-life of about 2–3 h and major routes of excretion were hepatobiliary and pulmonary routes. Altogether, these preliminary observations may suggest limited pharmacokinetic properties of plumbagin which result in inadequate systemic drug circulation. Furthermore, results of our previous studies show that both plumbagin and the crude extract of *P. indica* Linn. are relatively potent inhibitors of CYP450 [9, 10] and this is of concern for the potential herb-drug interactions when plumbagin or the extract containing plumbagin is to be co-administered with conventional drugs.

The aim of the present study was to further investigate the pharmacokinetics and toxicity of plumbagin using traditional pharmacokinetic study in rat model. In addition, we also investigated propensity of plumbagin to modulate mRNA expression and activities of the two inducible forms of hepatic drug metabolizing enzyme cytochrome P450 (CYP450), i.e., CYP1A2 and CYP3A11 in mice.

## Methods

### Chemicals

The authentic plumbagin (purity 98.2 %) was obtained from Apin chemicals Co. Ltd. (OX, UK). Tween-80, phenacetin, paracetamol, caffeine, omeprazole, 5-hydroxyomeprazole, nifedipine, oxidized nifedipine, ketoconazole, nootkatone, α-napthoflavone, and diazepam were purchased from Sigma-Aldrich (St. Louis, MO, USA). 1,4-Naphthoquinone was purchased from Wako Pure Chemical Industries, Co. Ltd. (Osaka, Japan). β-Nicotinamide adenine dinucleotide phosphate (reduced form) tetrasodium salt (NADPH) was purchased from Merck KGaA (Darmstadt, Germany). Pooled human liver microsomes (from 50 donors) were obtained from Gibco BRL Life Technologies (Grand Island, NY, USA). TRIzolTM reagent, SuperScript™ III Reverse Transcriptase kit, and SYBR Green Real-Time PCR Master Mixes were purchased from Invitrogen Life Technologies Inc. (Carlsbad, CA, USA). RQ1 RNase-Free DNase was purchased from Promega (Mannheim, Germany). MS grade water, methanol and acetonitrile were purchased from Yes-sci Co., Ltd. Isoflurane was purchased from Wako Pure Chemical Industries, Co. Ltd. (Osaka, Japan).

### Animals

Wistar rats (5 weeks of ages, weighting 200–210 g) of both genders were used for toxicity testing and pharmacokinetic investigation. Male ICR (Imprinting Control Region) mice (5 weeks of age, weighting 20–25 g) were used for investigation of the modulatory effect of plumbagin on CYP1A2 and CYP3A11. All animals were obtained from the National Laboratory Animal Centre, Thailand. Animal experiments were carried out in accordance with the *Guidelines for the Care and Use of Laboratory Animals*. They were housed under controlled light (12 h light and 12 h dark) and temperature (25 °C) in the animal house facility at Thammasat University. All were fed with a stock diet and water ad libitum. Approval of the study protocol was obtained from the Ethics Committee for Animal Research, Thammasat University, Thailand (certification number 005/2558).

### Toxicity testing

Plumbagin was dissolved with 20 % Tween-80 to obtain the desired concentrations. Wistar rats were fasting 2 h before administration of a single oral dose of the plumbagin. Animals were divided into eight groups of six (3 males and 3 females for each group). For the acute toxicity test, rats in each group were fed with plumbagin at a single oral dose of 500, 250, or 150 mg/kg body weight (1 mL); control group received a single oral dose of 20 % Tween-80 (1 mL). For the subacute toxicity test, rats in each group were fed with plumbagin at a daily dose of 100, 50, or 25 mg/kg body weight (1 mL) for 28 days; control group received a daily oral dose of 20 % Tween-80 for 28 days (1 mL).

General behavioral changes of each rat was observed continuously for 1 h after each dose, intermittently every 4 h, and thereafter over a period of 24 h [11, 12]. Animals were observed for up to 14 days for acute toxicity test and 28 days for subacute toxicity test, for any sign of toxicity (behavioral change related to central nervous, cardiovascular and gastrointestinal systems, body weight change, and water and food consumption). At the end of the observation period, all animals were sacrificed under ether anesthesia (isoflurane) and vital organs (liver, lung, kidney, heart, and spleen) were removed from all animals for gross and histopathological examination. Blood sample was collected from each rat through cardiac puncture for analysis of plasma chemistry (heparin as an anticoagulant) and blood hematology (EDTA as an anticoagulant).

### Pharmacokinetic study

Pharmacokinetic investigation was performed in 4 male Wistar rats (weighing 200–350 g) following a single oral dose of 100 mg/kg body weight plumbagin (1 mL). The control group received 20 % Tween-80 (1 mL). Blood samples (500 μL each) were collected from the tail vein of each rat at 0, 1, 2, 3, 5, 8, 24, 48, and 72 h after dosing. Plasma was separated from whole blood samples through centrifugation at 3000 × *g* for 10 min.

Concentrations of plumbagin in plasma and urine samples collected from all animals were measured using liquid chromatographic-mass spectrometry (LC-MS/MS) according to the previously described method with modifications [13]. The limits of quantification (LOQ) and detection (LOD) for plumbagin were 5 and 2 ng/mL plasma, respectively. Recovery of the compound in plasma after sample preparation procedure was between 82 and 92 %. The assay intra- and inter-day coefficients of variation (% CV) were 0.7–4.7 % and 3.9–8.3 %, respectively. In brief, plasma sample (100 μL) was mixed with 10 ng/mL honokiol (5 μL) and ethyl acetate (1 mL) and centrifuged at 12,000 × *g* for 15 min. The upper layer was transferred to a new tube and evaporated to dryness under nitrogen flow. The dried residue was reconstituted with 100 μL of the mobile phase and a 5 μL aliquot was injected onto the LC–MS/MS. The samples were collected from the metabolic cages at 24, 48, 72, 96, and 120 h. All were centrifuged at 1500 × g for 10 min and the supernatant (100 μL) was extracted with ethyl acetate (1 mL). The extract was dried and the residue was reconstituted with 100 μL of the mobile phase and a 5 μL aliquot was injected onto the LC–MS/MS.

The LC-MS/MS system used consists of Agilent 1100 Separation Module (Agilent Technologies, CA, USA) solvent and sample delivery, Av QTRAP 5500 Triple Quadrupole Mass Spectrometer (AB Sciex, Foster City, CA, USA) equipped with CID, and Multiple Reactions Monitoring (MRM). Mass spectrometric analysis was performed in the negative-ion mode (ESI). The following parameters of the turbo ion spray were used: ion spray voltage = −4500 V; the ion source gas 1 (GS1), gas 2 (GS2), curtain gas (CUR) and collision gas (CAD) were 50, 60, 25 and 5, respectively; and the desolvation gas (nitrogen) heated to 500 °C. Separation was performed using Hypersil gold reversed-phase C18 column (50 mm × 4.6 mm i.d., 5 μM: Thermo Scientific, Fremont, CA, USA) with the mobile phase consisting of a mixture of (A) water containing 0.1 % formic acid and (B) 100 % methanol, running with gradient mode at the flow rate of 0.6 mL/min at 25 °C (Table 1). On the basic of the full-scan mass spectra of each analyze, the most abundant ions were selected and the mass spectrometer was set to monitor the transitions of the precursors to the product ions as follows: m/z 187– >159 for plumbagin, and 265– >224 for honokiol (internal standard). The system was controlled by Analyst^TM^ software (AB Sciex, Foster City, CA, USA).

**Table 1:** HPLC condition for quantitative analysis plumbagin using gradient mobile phase consisting of (A) water containing 0.1 % formic acid and (B) 100 % methanol

Time (min)	0.1 % formic acid in water	100 % Methanol
0–2	60	40
2–2.5	20	80
2.5–7.5	20	80
7.5–8	60	40
8–10	60	40

Pharmacokinetic parameters were determined using a non-compartmental analysis using WinNonLin^TM^ software (version 6.3, Pharsights, Certara, USA). Concentrations of drugs lower than the limit of quantification (LOQ) were expressed as zero (undetectable). The C_max_ (maximum concentration); t_max_ (time of maximum concentration) were determined by direct inspection of the plasma concentration–time data. AUC (area under the concentration–time curve) from time 0 to 48 (AUC_0-48h_), and total AUC (AUC_0−∞_) were calculated using the trapezoidal rule. The extrapolated AUC from the last sampling time to infinity was estimated from Ct/elimination rate constant (λ_z_). λ_z_ was calculated from at least five concentration–time points of elimination phase. Apparent oral clearance (CL/F) was calculated as dose/AUC_0−∞_. Volume of distribution (V_z_/F) was calculated as CL/F/_z_, and the terminal half-life (t_1/2z_) was calculated as 0.693/λ_z_. Mean residence time (MRT) was calculated from the ratio of AUMC_0-α_)/AUC_0−∞_.

### Modulation of CYP450 activity

Mice were divided into four groups (3 male mice each). Mice in the treated groups were fed with plumbagin at a daily oral dose of 25, 12.5, or 6.25 mg/kg body weight (1 mL) for 28 days. The control group received a daily oral dose (1 mL) of 20 % Tween-80 for 28 days. At the end of the experiment period, all animals were sacrificed under ether anesthesia and livers were removed for investigation of the modulatory effect of plumbagin on hepatic CYP1A2 and CYP3A11.

#### Effect of plumbagin on CYP1A2 and CYP3A11 activities

The liver samples were rinsed with ice-cold, 0.9 %NaCl, homogenized in 50 mM sodium phosphate buffer (pH 7.4) containing 0.1 mM EDTA, and centrifuged at 9000 × g for 20 min. The supernatant was ultracentrifuged at 105,000 × g for 60 min, and the resulting microsomal pellets were suspended with ice cold 1.15 % KCl solution and ultracentrifuged at 105,000 × g for 60 min. The pellets were homogenized with 50 mM potassium phosphate (pH 7.4) containing 0.1 mM EDTA and 10 % glycerol. Protein concentrations were determined by Bradford protein assay [14] and the suspended microsomes were aliquoted and stored at −80 °C until use.

Phenacetin *O*-deethylation and nifedipine oxidation were used as selective markers for CYP1A2 and CYP3A11 activities, respectively [10]. The substrate concentration equivalent to the apparent K_m_ (Michaelis-Menten constant) of each individual CYP450 was used in the experiment. Final concentration of organic solvent in the incubation mixture was less than 1 %. The incubation conditions for determination enzyme activity to both CYP450 isoforms were according to the previously described methods [15–18].

The incubation mixture of 500 μL containing the selective substrate (20 μM phenacetin and 40 μm nifedipine for CYP1A2 and CYP3A11, respectively), mice liver microsomes (in 100 mM phosphate buffer pH 7.4) was pre-incubated at 37 °C for 5 min in a shaking water bath. The reaction was initiated by the addition of NADPH and further incubated for the specified periods. The incubation time (60 and 40 min for phenacetin *O*-deethylation and nifedipine oxidation, respectively) and protein concentration (0.3 mg/mL for both reactions) used were within the linear range for respective CYP450 activity. Each experiment was performed in triplicate. CYP1A2-mediated phenacetin *O*-deethylation and CYP3A11-mediated nifedipine oxidation reactions were terminated by the addition of 500 μL of cold acetonitrile. The internal standard (50 μL) caffeine (for CYP1A2) or diazepam (for CYP3A11) was added. Concentrations of paracetamol (metabolite of CYP1A2) and oxidized nifedipine (metabolite of CYP3A11) in the incubation mixtures were determined using HPLC with UV detection [8]. HPLC analysis was performed with HPLC system consisting of Spectra System HPLC with P4000 solvent delivery system, equipped with an AS3000 auto sampler, UV1000 detector, SN4000 controller (Thermo Finnigan, San Jose, CA, USA), and Chrome Quest software (version 4.0). Twenty microliters of each sample was injected onto the HPLC column for analysis. HPLC condition for determination of paracetamol consisted of C-18 reversed phase column (Thermo Hypersil Gold, 210 × 4.6 mm, 5 μm particle size), gradient mobile phase (a mixture of acetonitrile and distilled water) with an initial ratio of 10:90 at the flow rate of 1 mL/min, and UV detection at 240 nm. The calibration curve was prepared at the concentration range of 1–50 μM. HPLC condition for determination of oxidized nifedipine consisted of C-18 reversed phase column (Thermo Hypersil Gold, 210 × 4.6 mm, 5 μm particle size), mobile phase (a mixture of methanol and water at the ratio of 65:35 % *v/v*) with a flow rate of 1.0 mL/min, and UV detection at 270 nm. The calibration curve was prepared at the concentration range of 1–25 μM. The linearity of the calibration curve for each analytical assay for the CYP-mediated production of metabolite was demonstrated with the determination coefficient (r^2^) of greater than 0.995. Quality control (QC) samples were run in duplicate in each analytical batch at low, medium, and high concentrations. The criteria for acceptability was four out of six of the QC analyses to lie inside 100 ± 15 % of the nominal values.

#### Effect of plumbagin on CYP1A2 and CYP3A11 mRNA expression

The liver sample isolated from each mouse was rinsed with ice-cold, 0.9 % NaCl and total RNA was prepared using Trizol^TM^ reagent according to the manufacturer’s protocol (Invitrogen, Karlsruhe, Germany). Briefly, the sample was homogenized in TRIZOL reagent (1 mL) *per* 50–100 mg of tissue. The homogenized sample was incubated at 25 °C for 5 min and chloroform (0.2 mL/1 mL of TRIZOL reagent) was added. The sample was thoroughly mixed and incubated at 25 °C for 3 min. Following centrifugation (12,000 × *g* for 15 min, 2–8 °C), the sample was separated into a lower red phenol-chloroform phase, an interphase, and a colorless upper aqueous phase. The upper aqueous phase was transferred carefully into a fresh tube and RNA was separated by mixing with 0.5 mL isopropyl alcohol and incubated at 15–30 °C for 10 min. After centrifugation (12,000 × *g* for 10 min, 2–4 °C), the precipitated RNA was washed with 75 % ethanol (1 mL) and centrifuged at 7500 × *g* for 5 min (2–8 °C). The RNA pellets were dried and dissolved in 20 μL DEPC-treated water and the concentrations were determined using NanoDrop Spectrophotometry (NanoDrop Technologies, Wilmington, DE, USA). The RNA samples were treated with RQ1 RNase-Free DNase according to the manufacturer’s protocol (Promega, Mannheim, Germany) to degrade both double-stranded and single-stranded DNA.

The cDNA was prepared using the SuperScript™ III Reverse Transcriptase according to the manufacturer’s protocol (Invitrogen, Karlsruhe, Germany). Briefly, total RNA (10 pg–5 μg) and 1 μL of 50 μM oligo(dT)_20_ were incubated at 55 °C for 50 min. The reaction was inactivated by heating at 70 °C for 15 min and chilled on ice for 2 min. cDNA was synthesized in a total volume of 20 μL containing 5× First-Strand Buffer (250 mM Tris–HCl (pH 8.3), 375 mM KCl, and 15 mM MgCl_2_), 0.1 M DTT, 40 U RNaseOUT™ Recombinant RNase Inhibitor, 10 mM dNTPs, and 200 U Superscript III Reverse Transcriptase Rnase H (Invitrogen, Thermo Fisher Scienyific, NY, USA).

The forward and reverse primers for the selected genes used in the study are shown in Table 2 [19]. The platinum SYBRTM Green qPCR Supermix-UDG (Invitrgen, Carsbad, CA, USA) was used for real-time PCR (RT-PCR) analysis using iCycler IQ machine (BioRad Laboratories Inc., Hercules, CA, USA). The reaction mixture (25 μL) consisted of 50 ng/μL cDNA, platinum SYBRTM Green qPCR Supermix-UDG mixture, 10 μM forward primer, 10 μM reverse primer, and sterile double distilled water. The PCR cycles for *CYP1A2*, *CYP3A11* and *GAPDH* were as follows: denaturation at 95 °C for 10 min, followed by 40 cycles of amplification at 95 °C for 15 s, and annealing at 60 °C for 1 min. Each RT-PCR was performed in duplicate. Ct values (threshold cycle) which is the intersection between an amplification and threshold line was generated to reflect relative measure of the concentration of target in the RT-PCR reaction.

**Table 2:** Primer sequences used for determination of CYP1A2 and CYP3A11 mRNA expression

Gene	Gene accession number	Amplicon size (bp)	Primer sequence	Melt Temperature (°C)
CYP1A2	NM_009993	149	Forward: 5′-TGGTGGAATCGGTGGCTAAC-3′	82.50
			Reverse: 5′- GACCGGGAAGAAGTCCACTG-3′	
CYP3A11	NM_007818	121	Forward: 5′- ACCTGGGTGCTCCTAGCAAT-3′	80.50
			Reverse: 5′- GCACAGTGCCTAAAAATGGCA-3′	
GAPDH	NM_001289726	136	Forward: 5′- GGAGAGTGTTTCCTCGTCCC-3′	84.50
			Reverse: 5′- ATGAAGGGGTCGTTGATGGC-3′	

The delta-delta Ct method was used to calculate *CYP1A2* or *CYP3A11* gene expression level relative to control and the housekeeping gene GAPDH was used for normalization of *CYP1A2* or *CYP3A11* gene expression [19]. The delta-delta Ct calculation for the relative quantification of target gene was as follow:

$$ \begin{array}{l}\Delta \mathrm{C}\mathrm{t}(1)=\left[\mathrm{C}\mathrm{t}\left(CYP 1A 2\kern0.5em \mathrm{o}\mathrm{r}\kern0.5em CYP 3A 11\right)-\mathrm{C}\mathrm{t}(GAPDH)\right]\hfill \\ {}\Delta \mathrm{C}\mathrm{t}(2)=\Big[\mathrm{C}\mathrm{t}\left(\mathrm{control}\kern0.5em \mathrm{f}\mathrm{o}\mathrm{r}\kern0.5em CYP 1A 2\kern0.5em \mathrm{o}\mathrm{r}\kern0.5em CYP 3A 11\right)-\mathrm{C}\mathrm{t}\left(\mathrm{control}\kern0.5em \mathrm{f}\mathrm{o}\mathrm{r}\kern0.5em  GAPDH\right)\hfill \\ {}\Delta \Delta \mathrm{C}\mathrm{t}=\Delta \mathrm{C}\mathrm{t}(1)\Delta \mathrm{C}\mathrm{t}(2)\hfill \\ {}\mathrm{Relative}\kern0.5em \mathrm{expression}={2^{\Delta \Delta}}^{\mathrm{Ct}}\hfill \end{array} $$

where ∆Ct (1) = delta Ct of unknown sample, ∆Ct (2) = delta Ct of control, *CYP1A2* and *CYP3A11* are target genes, and GAPDH is a housekeeping gene.

### Statistical analysis

Statistical analysis was performed using SPSS version 16.0 (SPSS Inc., CO, USA). Distribution of all variables was assessed using Kolmogorov-Smirnov test for normality and results indicate non-normally distributed data. All quantitative variables are presented as median (interquartile range) for non-normally distributed data. Comparison of difference in quantitative variables between two groups and more than two groups was performed using independent Mann–Whitney *U* test and Kruskal-Wallis test (followed by pair-wise comparison by Mann–Whitney *U* test). Statistical significance level was set at α = 0.05 for all tests.

## Results

### Toxicity testing

All rats survived following a single oral dose of 150 mg/kg body weight plumbagin and 20 % Tween-80 (control). There was no visible sign of toxicity, or any change in water and food consumption and body weights of rats in both groups during the 14 days of observation. At higher doses (250 and 500 mg/kg body weight), plumbagin produced toxic signs and symptoms including anxiety, salivation, exhibited chewing, and agitation. Three (3/6) and all (6/6) of the animals in each group died, respectively. Histopathological examination of various organs (liver, kidney, heart, spleen, and lung) revealed no significant change in size and morphology in all animals receiving all dose levels of plumbagin. No gender-specific toxicity was observed at all dose levels.

For subacute toxicity test, all rats survived following a daily oral dose of 25 mg/kg body weight plumbagin and 20 % Tween-80 (control) for 28 days. There was no abnormality in behavior, sign of toxicity, nor changes in water and food consumption, as well as body weights in both groups during the 28 days observation period. Histopathological examination of various organs (liver, kidney, heart, spleen, and lung) revealed no significant change in size and morphology in animals of both group. There were no significant changes in blood chemistry and hematology of rats in both groups (Table 3A and B). Toxic signs and symptoms including anxiety, salivation, exhibited chewing and agitation were observed in 2/6 and 6/6 rats following the doses of 50 and 100 mg/kg body weight, respectively. All rats receiving 100 mg/kg body weight of plumbagin daily for 28 days died within 6–10 days, while two rats receiving 50 mg/kg body weight dosing died within 12–16 days. No rat died during dosing and after 28 days of dosing. No gender-specific toxicity was observed at all dose levels.

**Table 3:** Effects of plumbagin on (A) blood chemistry and (B) hematological parameters in serum of rats after daily oral administration at 25 mg/kg body weight for 28 days (3 males and 3 females for each group). Data are presented as median (interquartile range)

Parameter	Control	Plumbagin 25 mg/kg body weight
(A)		
Glucose (mg/dL)	248 (80)	230 (59)
BUN (mg/dL)	20.5 (6.5)	22.0 (7.5)
Creatinine (mg/dL)	0.50 (0.00)	0.50 (0.00)
Cholesterol (mg/dL)	68 (3)	73 (6)
Triglyceride (mg/dL)	106 (13)	110 (9)
AST (U/L)	100 (17)	101 (15)
ALT (U/L)	27 (10)	30 (11)
(B)		
RBC (×10^6^/μL)	8.27 (0.50)	8.16 (0.72)
Hemoglobin (g/dL)	14.9 (0.7)	14.4 (1.2)
Hematocrit (%)	47.9 (3.0)	47.8 (2.6)
WBC (×10^3^/μL)	9.10 (0.80)	9.40 (0.65)
Neutrophils (×10^3^/μL)	1.92 (0.11)	2.20 (0.63)
Eosinophils (×10^3^/μL)	0.50 (1.00)	0.00 (1.00)
Lymphocytes (×10^3^/μL)	7.41 (0.28)	7.59 (0.31)
Monocytes (×10^3^/μL)	0.00 (1.00)	0.00 (1.00)
Platelets count (×10^3^/μL)	870.00 (191.25)	885.50 (73.75)
Blood morphology	Normal	Normal

### Pharmacokinetic investigation

**Fig. 1:**
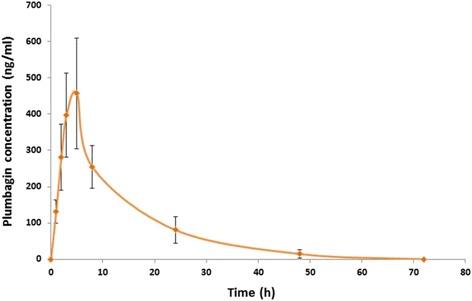
Median (interquartile range) plasma concentration–time profile of plumagin in male Wistar rats (*n* = 4) after a single oral administration of 100 mg/kg body weight of plumbagin

Plumbagin was detected in the urine samples up to 4 days (96 h) of dosing in all rats following a single oral dose of 100 mg/kg body weight of plumbagin. Median (interquartile range) of plumbagin excreted during 0–24, 0–48, 72, and 0–96 h were 389 (42), 258 (99), 15 (9) and 1.8 (0.3) μg, respectively. Total recovery of plumbagin during 0–96 h of dosing was only 3–5 %. The pharmacokinetic profile and parameters of plumbagin are presented in Fig. 1 and Table 4. Plumbagin was slowly absorbed after the oral dose reaching maximal plasma concentration at 5 h.

**Table 4:** The pharmacokinetic parameters of plumbagin after oral administration of 100 mg/kg body weight of plumbagin to healthy male Wistar rats [median (interquartile range), *n* = 4]

Parameter	Plumbagin
t_1/2_ (h)	9.63 (3.04)
t_max_ (h)	5.0 (0.0)
C_max_ (μg/mL)	0.46 (0.15)
AUC_0-48h_ (μg∙h/mL)	6.51 (1.16)
AUC_0−∞_ (μg∙h/mL)	6.71 (1.41)
MRT (h)	12.88 (2.49)
Vd/F (L/mg dose/kg body weight)	0.0022 (0.00)
CL/F (L/h/mg dose/kg body weight)	0.15 (0.3)

### Modulatory effect of plumbagin on CYP450

**Fig. 2:**
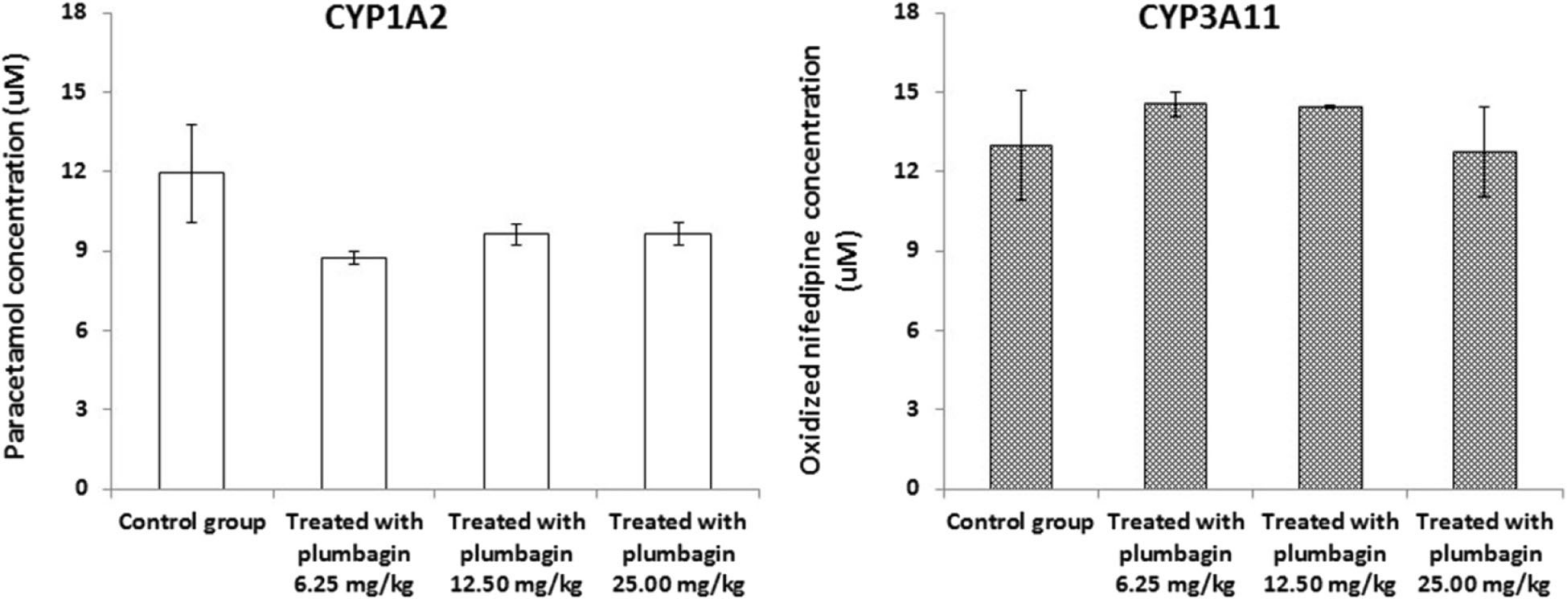
CYP1A2- and CYP3A11-mediated metabolism (generation of paracetamol and oxidized nifedipine, respectively) in mouse liver microsomes from the treated (induced by plumbagin for 28 days) and the control groups. Data are expressed as median (interquartile range) from 3 male mice

**Fig. 3:**
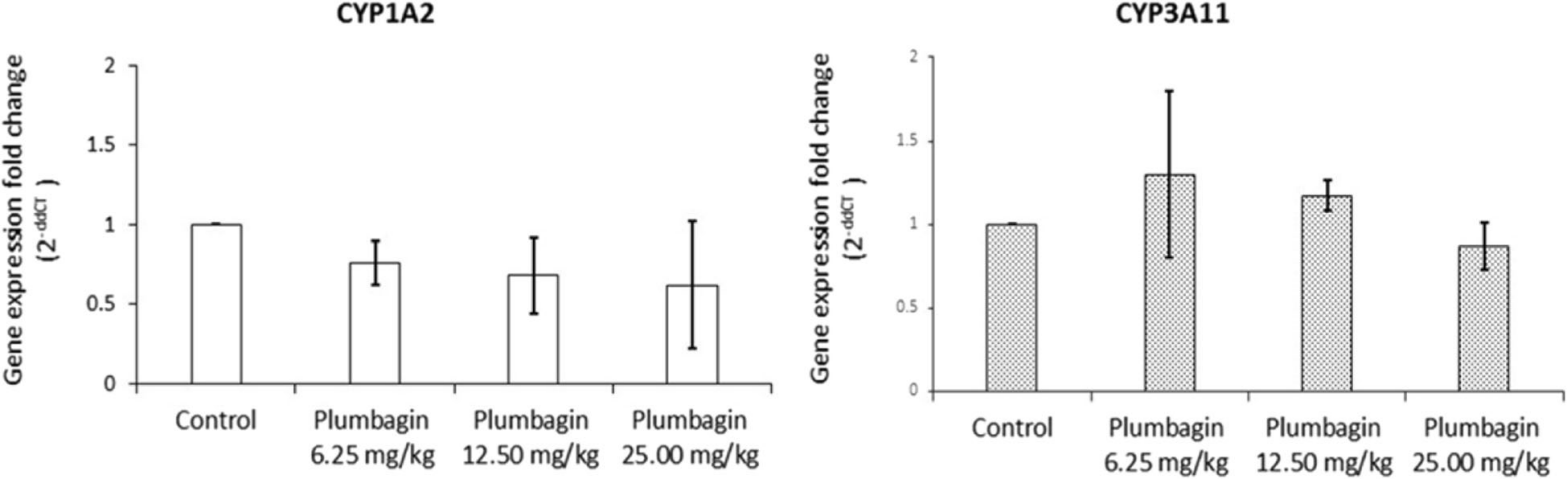
CYP1A2 and 3A11mRNA expression levels in mouse liver cells treated with 6.25, 12.5 and 25 mg/kg body weight for 28 days of plumbagin) and control groups. GAPDH was used as an internal control. CYP1A2 and 3A11mRNA expression levels were quantified by RT-PCR analysis in relation to GAPDH. Data are expressed as median (interquartile range) from 3 male mice

Figure 2 shows median (interquartile range) concentrations of paracetamol (CYP1A2-mediated phenacetin *O*-deethylation) and oxidized nifedipine (CYP3A11-mediated nifedipine oxidation) in liver microsomes prepared from mice following treatment with oral plumbagin at daily doses of 25, 12.5, and 6.25 mg/kg body weight and Tween-80 (control group) for 28 days. There was no significant change in the amounts of metabolites produced by both enzymes, although CYP1A2 activity tended to be reduced by plumbagin, particularly at the lowest dose level of 6.25 mg/kg body weight. These modulatory effects on CYP1A2 and CYP3A11 activities correlated well with the mRNA expression of both enzymes (Fig. 3).

## Discussion

The toxicity of plumbagin in rats was investigated in order to define the well tolerated dose of plumbagin for pharmacokinetic investigation. Selection of rat rather than mouse as a rodent species of choice is due to the possibility to collect larger blood volume required for quantitation of plumagin in plasma for pharmacokinetic analysis. The maximum tolerated doses of plumbagin in the acute and subacute toxicity testing were 150 (single oral dose) and 25 (daily doses for 28 days) mg/kg body weight, respectively. The 50 % lethal dose (LD_50_) of plumbagin for the acute and subacute toxicity testing were 250 and 50–100 mg/kg body weight, respectively. The dose selected for the pharmacokinetic study was a single oral dose of 100 mg/kg body weight, which is lower than the maximal tolerated dose (150 mg/kg body weight) to ensure that no rat died during the study. There was no significant change in hematological and blood biochemistry profiles of rats treated with maximal tolerated daily dose of 25 mg/kg for 28 days. In previous studies in mice however, various types of toxicity of plumbagin were reported. These included diarrhea, skin rashes, leukocytosis, increased serum phosphatase levels [20], hepatotoxicity [21, 22], and cardiotoxicity [23]. In a more recent study in mice [24], hepatotoxicity of plumbagin through unbalancing of the anti-oxidative system was observed, i.e., marked increased plasma ALT and AST levels, hepatic lipid peroxidation, and glutathione peroxidase activity; but decreased superoxide dismutase and catalase activities. Furthermore, by virtue of its similar chemical structure to vitamin K, plumbagin as a component of *Plumbago zeylanica*, was also suspected to affect blood hemostatis mechanisms. The platelet adhesion and coagulation was significantly decreased in the treated rats (daily doses of 2 mg/kg body weight) resulting in prolongation of bleeding time [25]. The discrepancies in such toxicity profiles observed in various studies could suggest species-specific toxicity of plumbagin and the extract of Plumbago spp. In addition, the dose level and duration given in the current study (25 mg/kg body weight for 28 days) may not be high enough to produce such toxicity. The maximal tolerated doses of plumbagin in the subacute toxicity testing in rats reported in the current study is similar to that reported in mice in our previous study (25 mg/kg body weight for 14 days), but the maximal tolerated dose in the acute toxicity testing in mice is relatively lower (100 mg/kg body weight). Results of both studies indicate low toxicity of plumbagin in both animal species. Nevertheless, hepatotoxicity as well as other toxic effects of plumbagin should be of great concern for future development of plumbagin as medicines. Dose ranging study to obtain information on safety window of effective and toxic doses should be carefully investigated. It is noted however that the ratios of plasma AST/ALT (>3:1) in both the control and treated rats were higher than the normal value reported in rats (2:1). Previous animal studies in Thailand using Wistar or Sprague Dawley rats (provided by the National Laboratory Animal Center of Thailand: NLACT) also reported the ratios of greater than 3:1 [26, 27]. Histopathological examination did not reveal any abnormality both in the control and treated groups. Plasma glucose level was also found to be relatively high (median values of 248 and 230 mg/dL in the control and treated rats, respectively) comparing with the normal range reported in Wistar rats (70–100 mg/dL) [28]. This hyperglycemic condition could be due to the influence of the volatile anesthetics isoflurane in impairing glucose-induced insulin release [29].

The pharmacokinetics study in Wistar rats revealed the median elimination half-life (t_1/2_), and mean residence time (MRT) of the plumbagin of about 9.6 and 5.0 h, respectively. These values were relatively short compared with the previously reported mean values of 17 and 22 h, respectively, in conscious freely moving Sprague–Dawley rats using liquid chromatography/tandem mass spectrometry [13]. The time to reach maximum blood concentration (t_max_) was about two times longer (5.0 *vs* 2.5 h). It was unexpected that only the species difference of rats used in both studies (Wistar *vs* Sprague–Dawley rats) could have had significant impact on the pharmacokinetics of plumbagin given by oral dosing. Although permeability across human intestinal membrane was reported to be moderate with no influence of efflux protein, oral bioavailability of plumbagin was shown to be relatively low (39 %) due to its limited biopharmaceutical properties such as high lipophilicity (log P 3.04) and insolubility in water [13]. Plumbagin was rapidly cleared from blood circulation and was only detected in systemic circulation up to 48 h. The time period until complete elimination in urine was 96 h, with extremely low recovery (3–5 % of the administered dose). This observation indirectly supports results from the previous study in mice using SPECT/CT imaging system which suggests hepatobiliary and pulmonary, rather than urinary excretion, as major routes of elimination of plumbagin [8]. ^99m^Tc-plumbagin complex was shown to be rapidly and extensively accumulated in livers and lungs of both healthy and infected mice as early as 5 min after dosing. Results of the in vitro study using human liver microsomes containing drug metabolizing enzymes supported the role of liver in the biotransformation of plumbagin, which well explained the high accumulation of ^99m^Tc-plumbagin complex in this organ [8]. Plumbagin is a volatile compound which is likely to be excreted by pulmonary route through exhaled air [25]. The pharmacokinetic profiles of plumbagin observed in this and other studies [13, 30] suggest that low systemic exposure might be a limiting pharmacokinetic factor that leads to inadequate drug concentration for antimalarial activity in animal models. This low systemic exposure may strongly affect potential development of plumbagin as an oral formulation. Various approaches have been applied to improve the oral bioavailability of plumbagin. These include preparation of liposomal formulation [31], and chitosan-based microspheres [30].

CYP450 represents a large family of proteins involved in the metabolism of drugs and other xenobiotics as well as endogenous compounds. As the major drug metabolizing enzymes in human liver, interactions between phytochemicals in herbal medicines and CYP450 are now well recognized because of their potential clinical and toxicological implications. The phytochemicals could act as substrates, inhibitors, or inducers of the CYP450 isoforms, which can lead to pharmacokinetic interactions with the co-administered drugs metabolized by the same CYP450 isoform [32, 33]. Various CYP450 isoforms including CYP1A2, CYP2A6, CYP2C, CYP2D6, CYP2E1, and CYP3A are differentially expressed in human liver (13, 4, 20, 2, 7 and 30 %, respectively) [34]. Inhibition of CYP450-mediated metabolism of the substrate drugs may result in toxic drug concentrations. On the other hand, induction of CYP450-mediated metabolism of the substrate drugs may result in inadequate therapeutic drug concentrations. Inhibition and induction of CYP450 enzymes by drugs and food or supplement sources is well documented [35, 36]. Our previous studies [9, 10] provide evidence for the inhibitory effect of plumbagin and *P. indica* Linn. on the three major hepatic CYP450 isoforms, i.e., CYP1A2, CYP3A4, and notably CYP2C19 (mean IC_50_ of 12.95 μg/mL vs. 1.39 μM, 6.43 μg/mL vs. 2.37 μM, and 4.71 μg/mL vs. 0.78 μM, respectively). In this study, plumbagin was not found to induce or inhibit the mRNA expression and enzyme activities of CYP1A2 and CYP3A11, although a trend of reduction of both mRNA expression and enzyme activity was observed with CYP3A11. Since the activities of both enzymes correlated well with their mRNA expression, this suggests that the changes in activities of both enzymes were results of reduced mRNA expression of both enzymes rather than the direct interference with the enzyme activities.

CYP1A2 is known to play a major role in the metabolism of pre-carcinogens and inhibitory effect of plumbagin to this CYP450 isoform may contribute only minor interaction with the co-administered drugs. On the other hand, this inhibitory effect would be expected to result in protection against cancers [37]. Mouse was selected as a rodent species for investigation of modulatory effect of plumbagin on CYP1A2 and CYP3A11. The major reason is that functional counterparts of almost all human genes exist within the murine genome. One-hundred and two and 57 putative functional CYP450 genes have been reported in mice and humans, respectively. Thirty-six orthologous pairs of CYP450 genes have been identified as having the potential for similar or identical functions in both mice and humans [38]. Human CYP3A4 and the murine homolog CYP3A11 are the major CYP450 regulated by pregnane X receptor. Although mouse CYP3A11 was identified to be the most similar human CYP3A subfamily with respect to catalytic activity [39], differences in inducibility and catalytic activity are possible. Since this CYP3A4 is the most important CYP450 isoforms with respect to drug metabolism in humans, extrapolating metabolism data from mice directly to humans should be performed with caution. With respect to CYP3A1A2, existing information provided strong support for the value and relevance of mouse CYP1A2-dependent metabolism to the corresponding metabolism in humans [40] in terms of catalytic activities and inhibitory profiles by selective inhibitor (furafylline).

## Conclusion

Plumbagin produced satisfactory toxicity profile in acute and subacute toxicity tests following oral dose administration in rats of both genders. Pharmacokinetics of plumbagin of being delayed absorption and short residence time in plasma could be the key factor contributing to the observed weak antimalarial activity in vivo. Plumbagin did not significantly modulate mRNA expression and thus, enzyme activities of both CYP1A2 and CYP3A11 in mouse livers. Therefore, potential interactions with co-administered drugs that are metabolized by both enzymes are unlikely. Further development of plumbagin for clinical uses in malaria and other diseases should be focused on modification of pharmaceutical formulation to improve pharmacokinetic properties. In addition, dose ranging study in animal models should be performed to obtain information on safety window of effective and toxic doses for extrapolation to human.
